# Children with Bronchopulmonary Dysplasia-Associated Pulmonary Hypertension Treated with Pulmonary Vasodilators—The Pediatric Cardiologist Point of View

**DOI:** 10.3390/children8050326

**Published:** 2021-04-22

**Authors:** Anna Migdał, Anna Sądel-Wieczorek, Edyta Ryciak, Alicja Mirecka-Rola, Grażyna Brzezińska-Rajszys, Małgorzata Żuk

**Affiliations:** 1 Department of Cardiology, The Children’s Memorial Health Institute, 04-730 Warsaw, Poland; a.sadel-wieczorek@ipczd.pl (A.S.-W.); a.mirecka-rola@ipczd.pl (A.M.-R.); g.brzezinska@ipczd.pl (G.B.-R.); m.zuk@ipczd.pl (M.Ż.); 2 Department of Neonatology, Pathology and Neonatal Intensive Care, The Children’s Memorial Health Institute, 04-730 Warsaw, Poland; e.ryciak@ipczd.pl

**Keywords:** pulmonary hypertension, children, bronchopulmonary dysplasia, sildenafil, vasodilators

## Abstract

Pulmonary hypertension in children with bronchopulmonary dysplasia (BPD-PH) significantly worsens the prognosis. Pulmonary vasodilators are often used in BPD-PH but the short-term outcome of treatment is not well described. The aim of this study was to evaluate BPD-PH children diagnosed beyond 36 weeks postmenstrual age treated with pulmonary vasodilators (sildenafil, bosentan, or both) and to assess the short and long-term effect of oral pulmonary vasodilators treatment. Twenty patients were included in the study. Cardiology evaluation (WHO-FC, NTproBNP, oxygen saturation, pulmonary to systemic pressure ratio PAP/SAP) was performed at diagnosis and after treatment initiation. In the majority of patients improvement in all evaluated factors was observed. No side effects of vasodilators were observed. PH resolved in 10 patients after a mean of 21.4 months of treatment. Six patients died. The number of poor prognostic factors commonly used to assess patients with pulmonary arterial hypertension (PAH) decreased significantly during BPD-PH treatment. The influence of BPD-PH perinatal risk factors on prognosis was considered but was not confirmed. In conclusion, the treatment of BPD-PH with pulmonary vasodilators was well tolerated and led to a clinical improvement with the possibility of discontinuation without recurrence of PH. Prognostic factors used in pediatric PAH risk stratification also seem to be useful in assessing treatment efficacy and prognosis in patients with BPD-PH.

## 1. Introduction

Pulmonary hypertension (PH) occurs in 17–43% of premature infants with bronchopulmonary dysplasia (BPD) and significantly worsens the prognosis. The mortality rate is remarkably higher (up to 50%) in a patient with bronchopulmonary dysplasia-associated pulmonary hypertension (BPD-PH) compared to BPD patients without PH [1,2,3].

Impaired lung development and abnormal vascular growth in premature infants result in a reduction of vascular density, alveolar hypoxia and lead to vascular remodeling, an increase in pulmonary vascular resistance (PVR), and right heart failure [4,5]. Although PH pathogenesis in BPD is multifactorial and its development may be influenced by various maternal, environmental, and genetic factors, some prenatal and postnatal variables are confirmed as PH risk factors [3,6].

Although guidelines for the early diagnosis and management of BPD-PH have been recently established [7,8], some infants may develop late BPD-PH—above 36 weeks postmenstrual age (PMA), despite even normal echocardiography at discharge.

The use of sildenafil is recommended [7,8] if there is no PH improvement despite oxygen saturation >92%, but there are still limited safety and efficacy data of vasodilators in this group of patients. Despite previous reports on the long-term outcomes of patients with BPD-PH treated with vasodilators [9,10,11,12,13,14], it is still unclear whether the prognosis is related to PH-specific therapy or spontaneous PH resolution with age. Recently published meta-analysis [15] included only a few publications on improving the estimated systolic PAP in a small heterogeneous group of patients (not only BPD, other forms of chronic lung diseases [10]) in whom sildenafil mostly was added to inhaled nitric oxide (iNO). Therefore, the aim of the study was to evaluate the clinical profile of patients diagnosed beyond 36 weeks PMA with late BPD-PH referred to pulmonary hypertension specialist for PH-specific therapy and to assess the short-and long-term effects of oral pulmonary vasodilator treatment.

## 2. Materials and Methods

Data of 20 patients with BPD and diagnosis of moderate to severe PH, referred to pulmonary hypertension specialist for PH specific therapy, were collected retrospectively from 2009 to 2015 and prospectively from 2016 to 2020. Since 2016, patients were treated in accordance with the 2016 BPD-PH recommendations [7,8] (addition of bosentan if no improvement after sildenafil, the possibility of empiric therapy without cardiac catheterization).

The diagnosis of BPD and its severity were defined using The National Institute of Child Health and Human Development (NICHD) criteria [16]. The PH was confirmed by echocardiography according to the current recommendation (2016) [7,8] based on the presence of tricuspid valve jet velocity more than or equal to 2.5 m/s, ventricular septal flattening, or bidirectional or right to left flow through the shunt. According to Kumar et al., pulmonary hypertension severity was classified as mild, moderate, and severe when the estimated right ventricle systemic pressure by echocardiography was <50%, 50–75%, and >75% of the systemic systolic pressure [17]. Cardiac catheterization was performed in patients diagnosed before 2016 and in selected cases (intracardiac shunts, patients over one year of age) in the prospective group.

WHO Functional Class (WHO-FC), N-terminal pro-brain natriuretic peptide (NTproBNP), oxygen saturation, a systolic pulmonary to systolic systemic pressure ratio (PAP/SAP) estimated on echocardiography from tricuspid regurgitation or pulmonary velocity acceleration time were evaluated at diagnosis and after treatment initiation within 1–3 months. The duration of treatment, follow-up to the end of data collection, clinical status, and available examination at last control were also assessed. Outcome parameters were resolution of BPD-PH confirmed by echocardiography and mortality.

Treatment efficacy and prognosis were assessed using available parameters classifying children with pulmonary arterial hypertension (PAH) to the high or low-risk group (according to the risk stratification tool [18]). The following high risk parameters were included: WHO-FC III-IV, NTproBNP >1200 pg/mL, right-to-left ventricle diameter ratio (RV/LV) >1.5, tricuspid annular plane systolic excursion (TAPSE) z-score <−3 [19], PAP/SAP >0.75.

Prenatal and postnatal risk factors for BPD-PH (gestational age, birth weight, small for gestational age, oligohydramnios, mechanical ventilation, high-frequency oscillatory ventilation, sepsis, hospitalization length of stay, BPD severity) [6] were investigated as possible prognosis risk factors.

Collected data contained results of examinations routinely performed in the management of patients with BPD-PH. Informed consent for all procedures and off-label treatment was obtained.

Due to the small size of the group, the study results are mostly descriptive. The data were presented as mean ± standard deviation and median (mean ± SD; median) for values with a normal distribution or median and interquartile range (median, Q1–Q3) in other cases. Qualitative variables were presented as a number and percentage of the study group N (%). If the size of the group was sufficient, statistical tests were performed (chi-squared test with the Yates’s correction, Student T-test for normally distributed data determined by the Shapiro–Wilk test, and Mann–Whitney or Wilcoxon test in other cases). Missing data were excluded by cases. The survival analysis was performed using the Kaplan–Meier survival function estimator. The results were shown in survival curves. To evaluate the significance of the difference between two or more survival functions a log-rank test was used. In all analyses, the level of significance *p* = 0.05 was adopted.

## 3. Results

### 3.1. Clinical Characteristic and Patient Management

The study group included 20 patients diagnosed with BPD-PH at the age ranged from 1.8 to 25.0 months. The mean age of BPD-PH diagnosis beyond 36 weeks PMA was 27 weeks (ranged from 1 to 104 weeks). All patients were discharged from the primary NICU, 4 patients were on noninvasive respiratory support (CPAP), one on mechanical ventilation at the time of diagnosis. PH was confirmed by echocardiography in all patients. Cardiac catheterization was performed in 11 patients due to pre- (*n* = 5) or post-tricuspid shunt (*n* = 2) or age at diagnosis >12 months (*n* = 4). Five of these patients were diagnosed before the BPD-PH 2016 recommendations. Congenital intracardiac shunt at diagnosis was confirmed in 11 pts: atrial septal defect (ASD) *n* = 8, ventricular septal defect (VSD) *n* = 3. In all patients, a shunt was not recognized as a primary cause but coexisting with PH. None of the patients had a diagnosis of pulmonary vein stenosis. A slight increase in one pulmonary vein flow velocity in one patient was observed, but it was not clinically significant. All of the patients received PH-specific therapy: 19 patients were treated with sildenafil. In 2 of them, without improvement after monotherapy, bosentan was added. One patient received bosentan as a monotherapy. The initial dose of sildenafil was 0.5 mg/kg every 8 h titrate to 1 mg/kg/dose. The starting dose of bosentan was 1 mg/kg twice daily followed by 2 mg/kg twice daily. No adverse effects after treatment initiation with vasodilators were observed. Seventeen patients with desaturation below 92% were on oxygen supplementation according to BPD-PH management recommendation [8]. The perinatal history and clinical characteristics of patients are shown in Table 1.

### 3.2. Short-Term Treatment Efficacy

**Figure 1:**
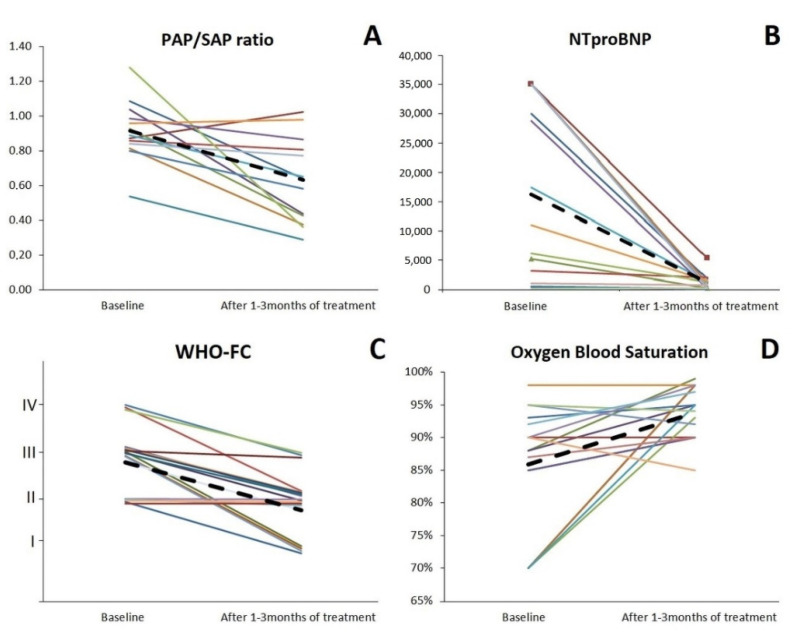
Changes in PAP/SAP (**A**), NTproBNP (**B**), WHO FC (**C**), and blood oxygen saturation (**D**) before and after 1–3 months of treatment. The black dashed lines represent the mean value. NTproBNP N-terminal pro-brain natriuretic peptide (pg/mL), PAP/SAP systolic pulmonary to systemic systolic pressure (ratio), WHO-FC World Health Organization Functional Class (classes), Oxygen Blood Saturation (%).

In the study group, 14 patients (70%) had severe PH, 6 patients (30%) moderate PH at diagnosis. After 1–3 months of PH-specific treatment, 11 patients improved: seven of them achieved PAP/SAP <50%—mild or absent PH. Seven patients had no change in the severity of PH, while two of them required treatment intensification (bosentan was added) (Figure 1A).

During treatment, normalization of NTproBNP level was observed in 7 patients, in 3 patients no significant improvement was achieved. Median NTproBNP level decreased significantly after starting treatment (10,500 vs. 619 pg/mL, *p* < 0.001) (Figure 1B).

Clinical condition changed after treatment initiation. Eleven patients changed the risk group from high (WHO-FC III-IV) to low (WHO-FC I-II) (Figure 1C). An improvement of saturation (mean 89 vs. 94% HbO_2_, *p* = 0.04) was observed, oxygen therapy was discontinued in three patients (Figure 1 D). Described changes are presented in Table 2.

### 3.3. The Effect of the Presence of a Shunt on Treatment and Follow-Up

The data of patients with a shunt and without shunt were compered (Table 3). All of the shunts at diagnosis were considered as coexisting, not causing PAH. ASD at the age of described patients does not lead to PH. Two infants with VSD had anatomically significant VSD but were not eligible for correction at the time of diagnosis (Qp:Qs < 1.5, PVR > 3), one patient had small VSD. Because of different pathophysiology, three patients with VSD were excluded from this comparison.

In children without shunt (*n* = 9), PH seemed to be diagnosed later than in ASD group (*n* = 8), but this difference was not statistically significant (median age 12.0 months (6.3–13.5) vs. 4.6 months (3.7–6.9); *p* = 0.07). A lack of shunt was associated with a greater number of BPD-PH development perinatal factors (median 5.0 (4.0–6.0) vs. 3.0 (1.75–3.25); *p* = 0.03). Both groups included patients with resolution of PH (no shunt *n* = 6, ASD *n* = 4) and patients who died (no shunt *n* = 2, ASD *n* = 1). Due to the small size of the groups, the number of deaths and the number of patients who discontinued treatment cannot be statistically compared. There were no significant differences between the groups in the other parameters tested. The efficacy of treatment in both groups was similar. During long-term follow-up, two ASD were surgically corrected.

### 3.4. Long-Term Follow-Up

Pulmonary vasodilators were discontinued if NTproBNP decreased to the normal level and echocardiography parameters did not indicate pulmonary hypertension. This occurred in 10 pts (50%) after a mean of 21.4 months of treatment (ranged from 4.7 to 40.7; median 16.6). The mean age at the time of PH resolution was 29.6 months (ranged from 9.3 to 53; median 28.8 months), mean age beyond 36 weeks PMA was 27.4 months (ranged from 6.4 to 51.3; median 26.3 months). No increase in NTproBNP level was observed 3 months after the end of treatment. No pulmonary hypertension signs were found in echocardiography performed 2–12 months after vasodilators discontinuation. The mean follow-up to the last cardiology assessment was 15.9 months (ranged from 2.0 to 53.1; median 9.5). No PH recurrence was noticed.

Four patients (20%) still require PH-specific medication (monotherapy), the duration of treatment range from 8.9 to 33.9 months.

Six deaths (30% of the study group) occurred after mean 3.9 (1.8–7.1; median 2.7) months of PH specific therapy: two due to sepsis, two in patients with VSD (one after PAB, one after VSD closure, deaths not related to pulmonary hypertension but a respiratory failure), two patients died suddenly at home. Further, 66% of deaths occurred before seven months corrected age (mean 7.7 ± 5; median 5.7 months).

### 3.5. Risk Factors and Survival Analysis

The comparison of two groups of patients: those who discontinued treatment in the long-term follow-up and those who died are presented in Table 4. Clinical status and PH severity parameters before and after 1–3 months of treatment were assessed. Patients who died were younger at the time of diagnosis and treatment initiation and had more severe PH (higher PAP/SAP) with higher WHO-FC class. Improvement in analyzed parameters (PH severity, mean PAP/SAP, WHO-FC, and level of NTproBNP) after treatment initiation was observed in both groups but seemed to be less marked in patients who later die than in surviving patients (NTproBNP level decreased less, WHO-FC class remained higher). Due to the small number of patients, statistical analysis was not performed.

**Figure 2:**
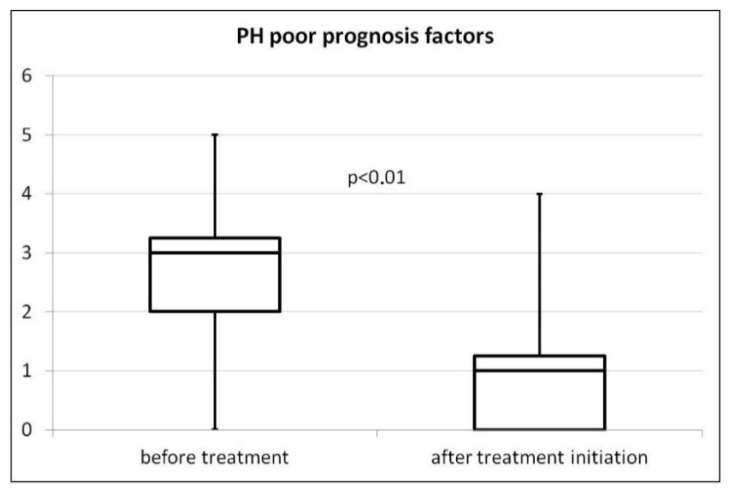
Pulmonary hypertension poor prognosis factors before and after 1–3 months of treatment with vasodilators (Box Plot chart, the box shows the first and third quartiles, the horizontal line in the middle is the median, the whiskers represent minimum and maximum of the data).

The number of high-risk prognostic factors recommended for risk assessment in children with PAH (only five parameters were available) in each patient was analyzed before and after 1–3 months of treatment. The median number of high-risk parameters at diagnosis was significantly higher than after treatment initiation (3.0 (2.0–3.25) vs. 1.0 (0–1.25), *p* < 0.001) (Figure 2). The influence of the number of high-risk factors on prognosis was analyzed. Although all groups of patients (death, end of treatment, still treated) had a similar median number of high-risk factors at baseline (3.0 vs. 2.5 vs. 2.5), those who later died appeared to have more median adverse prognostic factors after 1–3 months of treatment compared to the other groups (1.5 vs. 0.5 vs. 0.5). Because of the small group, statistical analysis was not performed.

The impact of nine known risk factors of PH development in BPD patients [6] on disease severity and prognosis was analyzed. Nineteen patients had at least 1 to 7 BPD-PH risk factors. The median number in the whole group was 3.5 (2.75–5.25). Ten patients had 0–3 risk factors (lower BPH-PH risk group-LR) and ten patients 4–7 (higher BPH-PH risk group-HR). In the LR group PH was diagnosed earlier (median age 3.7 (3.0–4.6) vs. 9.2 (5.4–12.5) months; *p* = 0.003) among children, that seem to be less mature (median age beyond 36 weeks PMA 7.0 (5.9–15.3) vs. 27.5 (15.2–42.2) weeks), although the difference is not statistically significant. Oxygen blood saturation at diagnosis also seemed to be higher (87.7 ± 10.0 vs. 84 ± 8.0% HbO_2_, NS) but without statistical significance. Eight out of 10 patients in the LR group were in III-IV WHO-FC. In the HR group, six patients had III-IV WHO FC. More patients with severe pulmonary hypertension were in the HR group (nine severe/one moderate vs. five severe/five moderate), and a significantly higher PAP/SAP ratio (0.94 ± 0.17 vs. 0.78 ± 0.17; *p* = 0.03) was observed. There was no difference in prognosis between both groups. The number of deaths was similar (LR—3 pts, HR—3 pts), and a similar number of patients completed treatment (LR—5 pts, HR—5 pts).

**Figure 3:**
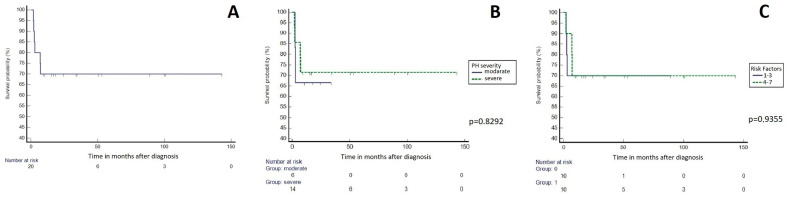
Kaplan-Meier survival curves for the whole group (**A**), and for subgroups divided by the PH severity (**B**), the and the number of perinatal risk factors (**C**).

Survival analysis was performed using recent data obtained at the end of the study. Such defined follow-up ranged from 1.8 to 143.1 months, median 17.5 (7.1–91.8). In entire group 1–3–6–9–12 months survival rate was respectively 100–80–70–70–70% (Figure 3A). There was no difference between estimated survival in patients with severe vs. moderate pulmonary hypertension (Figure 3B), and a high vs. a low number of perinatal risk factors (>3 vs. 1–3) (Figure 3C).

## 4. Discussion

### 4.1. Management of BPD-PH Patients Treated with Vasodilators

Our study consisted of 20 patients with moderate and severe PH referred to a cardiologist for PH-specific treatment despite maintaining optimal oxygen administration if needed. All patients were diagnosed beyond 36 postmenstrual weeks during standard cardiac care of patients with BPD (periodic examinations of BPD patients requiring oxygen therapy, increased oxygen demand). The small number of patients is a significant limitation, but the study was provided in a pediatric PH cardiology center where only selected patients are treated.

The age of the patients at the time of diagnosis and initiation of treatment varied, but 35% of them were over 1 year of age. In previous studies [9,10,11,12,13,14,20], most patients were younger, less than one year old. However, increased pulmonary artery pressure and pulmonary artery stiffness were recently reported in children [12] and young adults with a history of BPD in infancy [21]. This suggests that later detection of PH in children with BPD may be associated with aggravating factors, such as hypoxia or inflammation, during infection in children with increased vascular tone or undetected PH due to the absence of tricuspid valve regurgitation.

It is still discussed whether BPD-PH resolves spontaneously due to lungs development or with PH-specific therapy. Therefore, an early response to PH treatment should be analyzed. Several studies evaluated the long-term hemodynamic effect of vasodilator therapy on BPD-PH [10,11,12,14], but only a few showed an early improvement [22,23,24,25]. The recent meta-analysis of sildenafil use in preterm infants with BPD-PH (based on only five studies) [15] showed that treatment is associated with a reduction in estimated PAP in 69.3% of patients within 1–6 months, but in the majority of these patients, an oral vasodilator was added to iNO therapy, which could influence the effect of the therapy. Our study showed that BPD-PH oral vasodilator treatment during short-term observation leads to an improvement in PH severity in over 50% of patients. Moreover, an improvement in WHO-FC and a decrease in NTproBNP level after treatment was observed in our study. Only three studies to date evaluated the clinical effect of PH therapy on BPD-PH. Kadmon et al. [23] described significant reduction in Ross class. Slight improvement of respiratory severity score or oxygen requirements also were shown [22,25]. Although The Pediatric Pulmonary Hypertension Network (PPHNet) guidelines [8] suggest using serial BNP or NT-proBNP levels for monitoring disease severity and response to therapy, there is a distinct lack of data considering these biomarkers levels changes due to applied treatment in BPD-PH patients. Clinical evaluation using WHO-FC is not adapted to children with associated comorbidities common in premature babies. Moreover, dyspnea, which is the main symptom of WHO-FC, is not an uncommon or unexpected phenomenon in a child with BPD. There are no recommendations for assessing the functional status of these children. Perhaps Panama Pediatric Functional Class [26] dedicated to children would better reflect clinical assessment. The basic examination assessing the severity of PH in these patients is echocardiography, although not all patients have tricuspid regurgitation allowing to estimate systolic right ventricle pressure. Data from our study suggest that clinical assessment and NTproBNP may be additional parameters for assessing PH advancement. Taken together, our findings support a BPD-PH recommendation of vasodilator treatment in BPD-PH patients.

PH-specific treatment is complex in patients with a coexisting shunt. Although, Laggatta et al. [27] reported the presence of ASD as related to mortality in a large cohort of infants with BPD-PH, in our study the presence of ASD did not seem to affect the treatment effectiveness or survival rate, but a too small group could influence on the result. ASD appears to have been found in patients with BPD-PH diagnosed at an earlier age. This may result from higher rates of small insignificant shunts in infancy and the spontaneous closure of them with age. Similarly to del Cerro et al. [11], we observed that two patients with large ASD required surgical treatment in the follow-up during vasodilators therapy because of increased shunt volume due to a decrease in PVR. Regular cardiac assessment of these patients is necessary due to the possibility of hemodynamics changes and possible surgical closure.

Desaturation or worsening of ventilation parameters after the initiation of pulmonary vasodilators may result from an increased intrapulmonary shunting or ventilation-perfusion mismatch described by Nyp et al. [22]. In our study, no such effect was observed. Interestingly, the mean blood oxygen saturation significantly increased, and 3 patients who reached sat >92% no longer required oxygen therapy. It is not known whether the improvement seen with sildenafil is due to vascular effects or the possibility of improving lung function by a direct effect on bronchial smooth muscle or the amelioration of ventilation to perfusion ratio. In animal models, sildenafil not only decreased BPD-associated remodeling of the pulmonary vasculature and right ventricular hypertrophy, but also promoted alveolar development [28].

### 4.2. Efficacy of Treatment and Outcome

Our study group consisted of patients with severe and moderate PH qualified for vasodilator therapy following current recommendations. No further progression of pulmonary hypertension was observed after the start of treatment with mono- or dual therapy. No deaths related directly to the progression of pulmonary hypertension were confirmed, however, less improvement after treatment initiation seemed to be observed in patients who would later die. The mortality rate in infants with BPD-PH is high, ranging from 14–36% at six months and 34–47% two years after diagnosis of pulmonary artery hypertension [2,11,12]. Sepsis, respiratory failure, and comorbidities seemed to be the most common cause of death, not pulmonary hypertension. In a recent study, Arjaans et al. [13] observed the resolution of PH up to 2.5 years of age in 94% of survived patients, however 59% of them were on PH-specific therapy and patients with severe PH had a worse prognosis. Since the current guidelines recommend the treatment of moderate and severe PH-associated BPD with vasodilators, it was impossible to establish a control group to evaluate the effectiveness of treatment. Such a comparison can be made with a historical group without PH-specific treatment (Khemani et al. [2]) with a similar profile of patients (similar median gestational age, median birth weight, age of diagnosis, percentage of patients with systemic/suprasystemic PH, hemodynamic data). The percentage of death among patients without PH treatment and study group was similar (38% vs. 30%), but 87% of deaths in the Khemani group were caused by pulmonary hypertension. In our study, only two sudden deaths at home could be considered as possibly caused by PH, but only one of them concerned patients with severe PH. In the untreated group, the survival rate was significantly worse among patients with severe PH in contrast to our study group. Pulmonary hypertension severity improved in 89% of patients after a median of 9.8 months follow-up, but only 5 pts (11%) normalized pulmonary pressure, and 16 (38%) achieved <50% SAP. In our group, 7 pts (35%) achieved <50% SAP after just 1–3 months of treatment. However, the six-month survival rate was similar in both groups (70% vs. 64% in our group), later survival differed much more: two-year survival in patients without PH-specific treatment described by Khemani was 54% vs. 70% in our study. Comparison of our study with the historical control group suggests that treatment with vasodilators may be effective especially in patients with severe BPD-PH and influences on prognosis. As the ventilation strategy has changed over the years, this conclusion may be biased.

Despite the unfavorable prognosis described in the literature, discontinuation of vasodilators was possible among 50% of our study group after a median of 21.4 months of treatment without deterioration or pulmonary hypertension recurrence. Cohen et al. [14] also stated that BPD-PH is a form of pulmonary hypertension in which improvement allows for discontinuation of treatment in almost half of the patients but it has not yet been described why the pressure in the pulmonary artery normalizes and whether it results from an improvement in lung function or postnatal growth of the pulmonary vascular bed. Because pulmonary hypertension after sildenafil withdrawal recurrence was reported as temporary PH worsening during respiratory infections or progressive PH [11], these patients should be under further cardiologist observation. The optimal criteria and withdrawal time for sildenafil remain unclear and require further research.

### 4.3. Possible Risk Factors

Since the BPD-PH pathogenesis is multifactorial and still not fully understood, new factors affecting pulmonary angiogenesis are being investigated [29]. It is not known whether the accumulation of proven BPD-PH development factors influences the outcome. In our study, we did not observe worse outcomes in a group of patients with a larger number of these factors (≥4), although more severe PH was present among these patients.

Due to the lack of recommendations regarding risk assessment in patients with BPD-PH, we used the assessment for children with PAH. We observed that the number of selected factors can be useful in assessing treatment monitoring and prognosis, as in children with PAH. Such an analysis has not yet been conducted, and data from a larger group of patients are needed.

## 5. Study Limitations

The study data were collected partially retrospective and partially prospective and was conducted in years in which BPD-PH treatment guidelines were different. The study group consisted only of selected patients with severe and moderate BPD-HP referred to treatment with vasodilators, patients with mild BPD PH were not considered. Due to the lack of a control group, the effectiveness of the treatment was assessed in comparison with the historical group not receiving PH-specific treatment. Because of the small size of the group, the comparison between survivors and non-survivors could not be done using statistical methods. The results of this study concern a small group of patients, and the extension of the BPD-PH population requires further studies on a larger group of patients.

## 6. Conclusions

Treatment of severe and moderate PH associated with BPD with pulmonary vasodilators was well tolerated and led to clinical improvement in most patients as well as improvement and even normalization of pulmonary hypertension parameters after 1–3 months of treatment. The treatment used in accordance with current recommendations enabled the discontinuation of vasodilators in half of the patients without a recurrence of pulmonary hypertension in the study period. Selected prognostic factors, the same as in PAH risk stratification in children, also seem to be useful in assessing treatment efficacy and prognosis in patients with BPD-PH. Mortality in patients with PBD-PH is still high, particularly during the first six months after diagnosis of PH, but treatment with vasodilators may reduce the mortality caused by pulmonary hypertension.

## Figures and Tables

**Table 1:** Perinatal history and clinical characteristics.

Patients’ Characteristics		Patients *n* = 20
Age at diagnosis (months)		5.0 (3.2–12.4)
Age at diagnosis beyond 36 weeks PMA (weeks)		15.2 (6.7–41.2)
Gestational age at birth (weeks)		27.7 ± 4.26; 26 (24–30)
Birth weight (g)		735 (675–920)
**BPD-PH risk factors according to Nagiub et al. [6]**		
BPD	mild	3 (15%)
	moderate	3 (15%)
	severe	14 (70%)
Ventilation duration (non-CPAP) >60 days		11 (55%)
Hospitalization days >90		19 (95%)
Oligohydramnios <2 cm		4 (20%)
Birth weight <600 g		2 (10%)
High-Frequency Oscillatory Ventilation usage		8 (40%)
Small for gestational age <3rd centile		9 (45%)
Sepsis >30 days antibiotics		5 (25%)
Gestational age <25 weeks		6 (30%)
**Other comorbidities**		
Necrotizing enterocolitis		5 (25%)
Intraventricular hemorrhage (grade III or IV)		4 (20%)
Retinopathy of prematurity		10 (50%)
Gastroesophageal reflux disease		5 (25%)
Cholestasis		2 (10%)
Down Syndrome		3 (15%)
**Hemodynamics**		
Echocardiography	PAP/SAP	0.90 ± 0.17; 0.88
Cardiac catheterization (*n* = 11)	mean PAP (mmHg)	39.5 ± 17.1; 36.0
	mean PAP/mean SAP (mmHg)	0.78 ± 0.30; 0.71
	PCWP	8.0 ± 3.5; 7.0
	Qp:Qs	1.24 ± 0.36; 1.09
	PVR/SVR	0.62 ± 0.38; 0.52
	PVRi (Wood units x m^2^)	8.4 ± 5.0; 7.0
Shunts	PDA ligation in neonatal period	7 (35%)
	ASD	8
	VSD	3

Data are shown as *n* (%), mean ± SD; median or median (interquartile range). ASD atrial septal defect, BPD-PH bronchopulmonary dysplasia associated pulmonary hypertension, CPAP continuous positive airway pressure, mPAP/mSAP mean pulmonary artery to mean systolic arterial pressure, PAP/SAP systolic pulmonary to systolic systemic pressure ratio, PDA patent arterial duct, PCPW pulmonary capillary wedge pressure, PH pulmonary hypertension, PMA postmenstrual age, PVRi indexed pulmonary vascular resistance, PVR/SVR pulmonary to systemic vascular resistance ratio, Qp:Qs pulmonary to systemic flow, VSD ventricular septal defect.

**Table 2:** Clinical status and PH severity parameters at diagnosis and after 1–3 months of treatment.

		Baseline *n* = 20	After 1–3 Months of Treatment *n* = 18	*p* **
PH severity	Severe	14 (70%)	5 (27%)	
	Moderate	6 (30%)	6 (33%)	
	Mild	0	6 (33%)	
	Absent	0	1 (5%)	
PAP/SAP *		0.9 ± 0.17; 0.88	0.61 ± 0.24; 0.63	0.02
WHO-FC	I–II	6 (30%)	14 (78%)	0.003
	III–IV	14 (70%)	4 (22%)	
NTproBNP (pg/mL)		15,888 ± 14,598; 10,500	1052 ± 1277; 619	0.001 ***
Blood oxygen saturation (%HbO_2_)		86 ± 9; 89	94 ± 4; 94	0.04
SAT	<90% HB02	10 (50%)	1 (6%)	0.003
	>90% HB02	10 (50%)	17 (94%)	
Oxygen supplementation		17 (85%)	12 (66%)	NS

Data are shown as *n* (%), mean ± SD; median. * only in patients in whom PAP/SAP estimation was possible (*n* = 18), ** statistical analysis for 18 patients with complete data, *** NTproBNP measurements before (*n* = 16), and after (*n* = 18) treatment, statistical analysis done for possible 16 measurements, NTproBNP N-terminal pro-brain natriuretic peptide (pg/mL), PAP/SAP systolic pulmonary to systemic systolic pressure (ratio), SAT blood oxygen saturation, WHO-FC World Health Organization Functional Class (classes).

**Table 3:** Comparison of patients with presence of a coexistent shunt (ASD) with patients without a shunt at diagnosis and after treatment initiation.

		ASD *n* = 8			No Shunt *n* = 9		
		Before Treatment	After Treatment Initiation	*p*	Before Treatment	After Treatment Initiation	*p*
SAT (%HbO_2_)	Median (inter-quartile range)	91 (85–92.75)	97.5 (94.75–98)	0.03	87 (80–90)	93 (90–95)	0.03
WHO-FC		3.0 (2.75–3.0)	2.0 (1.0–2.0)	<0.01	3.0 (2.0–3.0)	2.0 (2.0–2.0)	<0.01
NTproBNP (pg/mL)		28,762 (6042–35,000)	564 (246–1192)	0.01	12,441 (2826–20,036)	470 (256–952)	0.02
**Echocardiography Parameters**							
PAP/SAP	Mean ± SD; median	0.82 ± 0.13; 0.84	0.58 ± 0.22; 0.58	0.01	0.93 ± 0.19; 0.89	0.65 ± 0.23; 0.63	0.04
TAPSE (Z score)		0.13 ± 1.65; 0	0.08 ± 1.36; 0	NS	−2.62 ± 3.44; −2.11	−1.37 ± 1.73; −1.75	NS
LVDd (Z score)		−2.4 ± 0.9; −2.4	−1.3 ± 1.1; −0.7	0.02	−1.97 ± 1.31; −2.11	−0.10 ± 0.71; −0.08	0.01

LVDd-left ventricular diastolic diameter, NTproBNP N-terminal pro-brain natriuretic peptide (pg/mL), PAP/SAP systolic pulmonary to systemic systolic pressure (ratio), SAT blood oxygen saturation, TAPSE tricuspid annular plane systolic excursion (Z score), WHO-FC World Health Organization Functional Class.

**Table 4:** Clinical status and PH severity parameters at baseline and after 1–3 months of treatment among patients who discontinued treatment or died in a long-time follow-up.

Follow-Up		Baseline		After 1–3 Months of Treatment	
		End of Treatment *n* = 10	Death *n* = 6	End of Treatment *n* = 9 **	Death *n* = 5 ***
Age at diagnosis (months)		6.4 (4.6–12.2)	3.2 (3.0–4.5)		
Age at diagnosis beyond 36 weeks PMA (weeks)		3.9 (2.1–9.3)	1.9 (1.4–3.2)		
BPD-PH risk factors		4.0 (3.0–5.0)	4.0 (3.0–6.0)		
PH severity	Severe	7	4	2	1
	moderate	3	2	3	2
	Mild			4	1
	Absent				1
PAP/SAP *		0.87 ± 0.17; 0.87	1.02 ± 0.19; 0.97	0.55 ± 0.24; 0.44	0.48 ± 0.27; 0.41
WHO-FC	I–II	5	0	9	2
	III–IV	5	6	0	3
NTproBNP (pg/mL)		20,190 ± 17,095; 30,072	13,124 ± 10,246; 10,000	1113 ± 1689; 470	1249 ± 715; 1318
Oxygen blood saturation (%HbO_2_)		84 ± 10; 88	86 ± 10; 86	94 ± 4; 95	93 ± 3; 92

Data are shown as *n*, mean ± SD; median or median (interquartile range). * only in patients in whom PAP/SAP estimation was possible, ** No available results of follow up examination in one patient, *** One patient died before follow-up examination. BPD-PH bronchopulmonary dysplasia associated pulmonary hypertension, NTproBNP N-terminal pro-brain natriuretic peptide (pg/mL), PAP/SAP systolic pulmonary to systemic systolic pressure (ratio), PH pulmonary hypertension, WHO-FC World Health Organization Functional Class.

## Data Availability

Data presented in this study are available on reasonable request to the corresponding author. Data are not publicly available because they report private information about participants.
